# Past and present of the enigmatic genus *Lanayrella* Salvador & Cunha, 2020 (Gastropoda: Cephalaspidea) in the southwestern Atlantic Ocean

**DOI:** 10.7717/peerj.21120

**Published:** 2026-04-23

**Authors:** Javier Di Luca, Guido Pastorino

**Affiliations:** Laboratorio de Ecosistemas Marinos (LEMar), Lab. 138, Museo Argentino de Ciencias Naturales ‘Bernardino Rivadavia’, Ciudad Autónoma de Buenos Aires, Argentina

**Keywords:** Fossil, Cylichnidae, Microgastropods, Systematic, Taxonomy, Patagonia, Magellan region, Toledonia, Bogasonia, Diaphanidae

## Abstract

The genus *Lanayrella* currently comprises two recent, minute gastropods species, *Lanayrella vagabunda* and *L. ringei*, both known exclusively from the Magellanic Region. The genus was originally assigned to Acteonidae because it shares with *Acteon* a relatively wide and thick shell with a moderately elevated spire, a sculpture dominated by spiral elements, and a strong columellar fold. However, it lacks a distinct proto/teleoconch boundary, a diagnostic feature of *Acteon*. Here, *L. ringei* is studied for the first time based on shell morphology and gross anatomy, which reveal as key characters a cephalic shield divided into two lobes with lateral tentacular pads and the absence of an operculum. These features strongly suggest that *Lanayrella* belongs among cephalaspidean gastropods rather than within Acteonidae, a “lower” heterobranch taxon. Furthermore, both shell and radular morphologies are comparable to those of certain species of *Toledonia* and *Bogasonia,* leading to *Lanayrella bullata* n. comb. here proposed. Specimens from the Monte León Formation (lower Miocene) of southern Argentina are herein reported as *L. vagabunda* representing the first fossil record of the genus. This finding indicates that *Lanayrella* is part of an ancient lineage of highly distinctive gastropods whose presence in southern South America can be traced back at least 20 million years.

## Introduction

The genus *Lanayrella* Salvador & Cunha, 2020 was described within the family Acteonidae d’Orbigny, 1842 to include *Lanayrella vagabunda* (Mabille, 1885) and *L. ringei* (Strebel, 1905), two very small, recent species known only from empty shells collected in southern South America (Salvador & Cunha, 2020). These species point to *Acteon* Montfort, 1810 because they share relatively wide and thick shells with a moderately elevated spire, a pitted sculpture pattern consisting of spiral furrows crossed by fine growth lines, and a strong columellar fold; however the protoconch morphology is diagnostic. It lacks a distinct boundary with the teleoconch and according to Salvador & Cunha (2020) contrasts with *Acteon* and is also comparable with *Toledonia* Dall, 1902.

Marcus (1976) examined the anatomy of *Toledonia bullata* (Gould, 1847), based on specimens identified by Powell (1951) as *Acteon bullatus*. She demonstrated the absence of an operculum, and the presence of a radula with a prominent cusped rachidian tooth, among other anatomical features that supported its placement in *Toledonia* rather than in Acteonidae. Owing to the shell similarities between *T. bullata* and the two species currently placed in *Lanayrella*, Marcus (1976) considered all three species to belong to *Toledonia*.

The aim of the present study is to clarify the systematic position of the genus *Lanayrella* within heterobranch gastropods. To this end, the gross anatomy of *L. ringei* is examined for the first time. In addition, fossil specimens attributable to *L. vagabunda* are reported from the Monte León Formation (lower Miocene) in southern Argentina.

## Materials and Methods

### Materials studied, methodology employed and institutional repositories

Recent material was either collected manually or during cruises to the Magellanic Region conducted as part of the “Campaña Antártica de Verano” aboard R/V Puerto Deseado. Bottom samples were taken using a dredge net (two mm mesh), fixed in 5% formalin solution on board, and later transferred to 70% ethanol. Fossil material comes from six bulk samples of five L collected from shell beds at the Cabeza de León and Monte Entrada localities, situated within or near the boundaries of Monte León National Park. Samples were washed with diluted H_2_O_2_ and sieved following the standard procedures for processing Foraminifera and small mollusks described by Beu & Maxwell (1990). Both recent and fossil specimens were sorted and examined under a Leica MZ 95 stereoscopic microscope. They were photographed using a Zeiss Discovery V20 stereoscopic microscope and/or illustrated with a Phillips XL 30 and Zeiss Gemini 360 scanning electron microscopes (SEM), all housed at the Museo Argentino de Ciencias Naturales (MACN). Radulae were extracted by dissolving the soft parts in commercial bleach. Specimens are deposited at the Invertebrate (MACN-In) and Paleoinvertebrate (MACN-Pi) collections of the MACN. Type materials studied are housed at Muséum national d’Histoire naturelle (MNHN), Paris, France and Zologisches Institut und Zoologisches Museum der Universität (ZMH), Hamburg, Germany.

### Geological framework of the fossil materials

The fossils described herein were collected from shell beds at the top of the Punta Entrada Member of the Monte León Formation (Bertels, 1970; Bertels, 1980). These shell beds occur within loose or very poorly cemented sandstones, lithologies interpreted as part of the generally regressive sedimentary succession represented by the Monte León Formation. The fossil concentrations are parautochtonous and contain a diverse, abundant, and well-preserved macrofauna (Ihering, 1907; del Río & Camacho, 1998; del Río, 2004a; del Río, 2004b; Griffin & Pastorino, 2005; Griffin & Pastorino, 2006; del Río & Martínez, 2006 and references therein). The Cabeza de León and Monte Entrada localities were considered in this study; schematic sections of both sites were provided by Parras & Griffin (2009) and Griffin & Pastorino (2012).

The Monte León Formation has been dated as Chattian to Rupelian (Oligocene), based on its foraminiferal content (Bertels, 1970; Bertels, 1975). Subsequently, Náñez (1988) proposed an upper Oligocene–lower Miocene age for the unit, whereas Barreda & Palamarczuk (2000) considered it lower Miocene based on palynological data. Parras, Dix & Griffin (2012) assigned the Monte León Formation an entirely lower Miocene age (Aquitanian to early Burdigalian) based on 87Sr/86Sr dating of oyster, pectinid and brachiopod shells, with ages ranging from 22.12 Ma (+0.46, −0.54) at the base, to 17.91 Ma (+0.38, −0.4) at the top of the unit.

## Results

**Table:** 

Class Gastropoda Cuvier, 1795
Subclass Heterobranchia Burmeister, 1837
Order Cephalaspidea Fischer, 1883
Genus *Lanayrella*Salvador & Cunha, 2020

Type species: *Tornatella vagabunda* Mabille, 1885 by original designation.

Included species: *Lanayrella bullata* (Gould, 1847) n. comb., *L. vagabunda* (Mabille, 1885); *L. ringei* (Strebel, 1905).

**Table:** 

*Lanayrella ringei* (Strebel, 1905)
Figs. 1 and 2

*Actaeon ringei* Strebel, 1905: 576, pl. 22, figs. 31, 31a-b; Carcelles, 1950: 67; Carcelles & Williamson, 1951: 310

*Acteon ringei* Strebel, 1905—Powell, 1960: 161

? *Toledonia ringei* Strebel, 1905—Marcus, 1976: 29, fig. 32

*Acteon vagabundus* (Rochebrune & Mabille, 1885)—Castellanos, Landoni & Dadon, 1993: 8 (in part), fig. 5; Forcelli, 2000: 116 (in part), fig.

*Toledonia vagabunda* (Rochebrune & Mabille, 1885)—Forcelli & Narosky, 2015: 206, pl. 7, fig. 18

*Lanayrella ringei* (Strebel, 1905)—Salvador & Cunha, 2020: 1015, fig. 2

Type material: three syntypes (ZMH 12833) from Strait Le Maire, Tierra del Fuego, Argentina.

Examined material: Recent: one syntype (photographs); Punta Peña, Puerto San Julián, Santa Cruz, intertidal (MACN-In 44876: 2 an.); Ushuaia, Tierra del Fuego (MACN-in 44877: 1 sh.).

**Figure 1:**
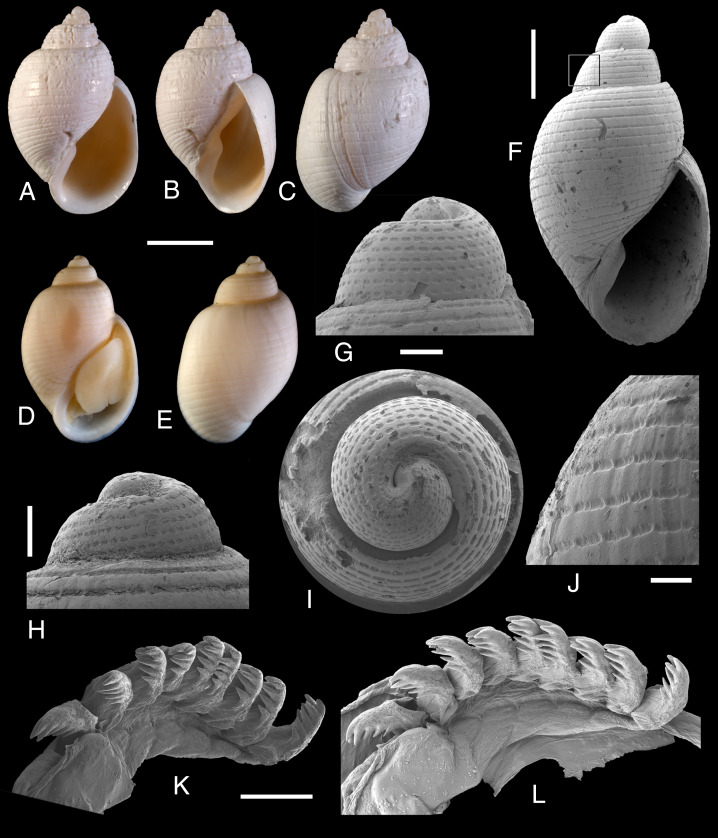
*Lanayrella ringei* (Strebel, 1905). (A–E, H, K, L) Specimens from Punta Peñas, Santa Cruz Province (MACN-In 44876). (F, G, I, J) Specimen from Ushuaia, Tierra del Fuego Province (MACN-In 44877). (A–C) Three views of the shell. (D, E) Two views of the shell. (F) View of the shell at SEM. (G–I) Three views of the apical whorls at SEM. (J) Detail of the sculpture at SEM. (K, L) Two views of the radula at SEM. Scale bars: (A–E) two mm. (F) one mm. (G, I) 200 µm. (H) 200 µm. (J) 200 µm. (K, L) 50 µm.

Description: Shell (Figs. 1A–1F) small (up to 8.2 mm in length), thick, ovate, with 4½whorls. Spire medium-sized about 20% of total length. Apical whorl (Figs. 1G–1I) tilted, with slightly sunken nucleus; proto-teleoconch limit not visible. Teleoconch whorls convex, suture impressed to slightly channeled. Sculpture (Fig. 1J) consisting of narrow, incised spiral furrows, regularly arranged over the entire shell surface: up to 11 on the apical whorl, five to eight on spire and 20 to 27 on the last whorl. Furrows interrupted by few, very thin, slightly prosocline growth lines; intersections between furrows and lines produce a regular pitted pattern, more evident on early whorls where more lines are also present (Figs. 1G–1I). Aperture large, somewhat elongate; lip thin and sharp; columella strongly curved, abapically arched, with a single, strong fold; callus narrow. Periostracum thin, translucent, and persistent.

Radula (?1:1:?1) (Figs. 1K, 1L) rachidian tooth prominent, narrow, triangular, with a slightly retracted central cusp and up to four pairs of lateral cusps. Lateral teeth, unclear, very weak plate-like and relatively large.

**Figure 2:**
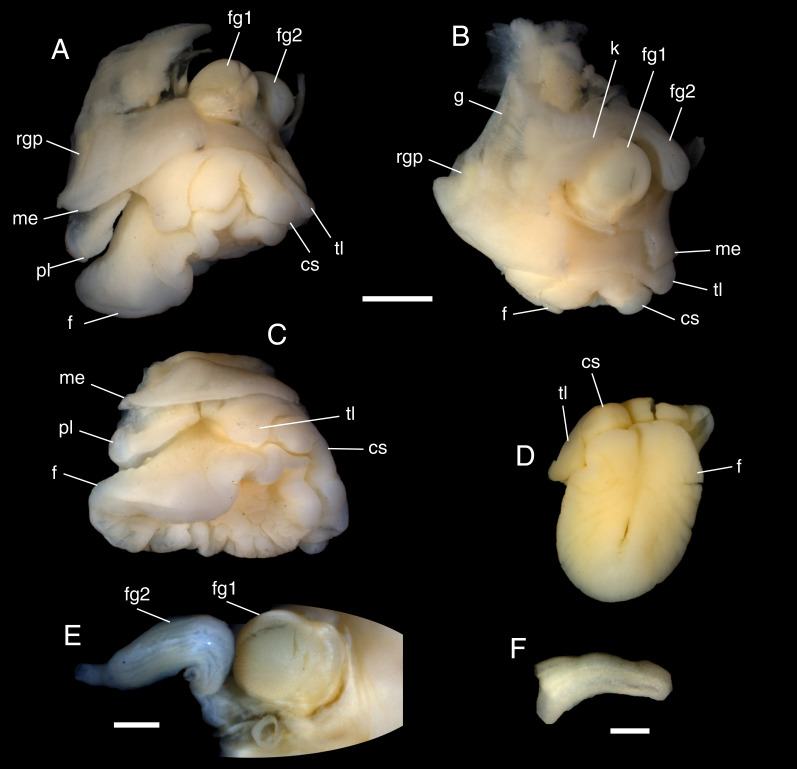
*Lanayrella ringei* (Strebel, 1905) from Punta Peñas, Santa Cruz Province (MACN-In 44876). (A–C) Three views of the relaxed soft parts. (D) View of the retracted soft parts. (E) View of the females organs. (F) View of the penis. Scale bars: (A–D) one mm. (E) 500 µm. (F) 300 µm. Refs: *cs*, cephalic shield; *f*, foot; *fg1, 2*, female reproductive system glands; *g*, gill; *k*, kidney; *me*, mantle edge; *pl*, posterior lobe; *rgp*, posterior repugnatory gland; *tl*, tentacular lobes.

Soft parts (Figs. 1D; 2). In fixed specimens, soft parts are whitish and can retract at least as far as the aperture (Fig. 1D). The head (Fig. 2A) is broad, bearing a cephalic shield sharply divided in two central, relatively large lobes. Sessile eyes are located at the base of the shield. Posterior to the cephalic shield, a pair of large, rectangular tentacular lobes with a broad basal connection is present. The foot is relatively large when extended (Figs. 2A–2C), suboval in shape, and slightly notched anteriorly. On the ventral surface, the ducts of the pedal glands are visible; they open into a deep median furrow that fades towards the posterior margin (Figs. 1D; 2D). The mantle bears a large posterior lobe (Figs. 2A, 2C). Two large, yellowish repugnatory (?) glands are present: one situated anteriorly on the left side and the other posteriorly towards the right-dorsal side. An elongated kidney extends from the right towards the median region of the outer surface, and a similarly elongated gill lies posteriorly. The digestive system includes a strong pharynx and digestive glands occupying the spire whorls. The reproductive system is hermaphroditic (Figs. 2E, 2F). The female gonopore opens on the posterior right side of the last whorl, whereas the male gonopore is located near the right eye. A groove runs obliquely along the right side of the foot towards the female gonopore. A prominent gland, probably involved in the production of capsules and intracapsular nutritive fluid, lies on the posterior right side of the last whorl; this gland connects to a female duct that runs to the gonopore opening (Fig. 2E). The penis (Fig. 2F) is retractile, somewhat flattened, and apically enlarged.

Distribution: Beagle Channel (type locality), Tierra del Fuego province; Puerto San Julián, Santa Cruz province (this work); Buenos Aires province (Forcelli & Narosky, 2015).

**Figure 3:**
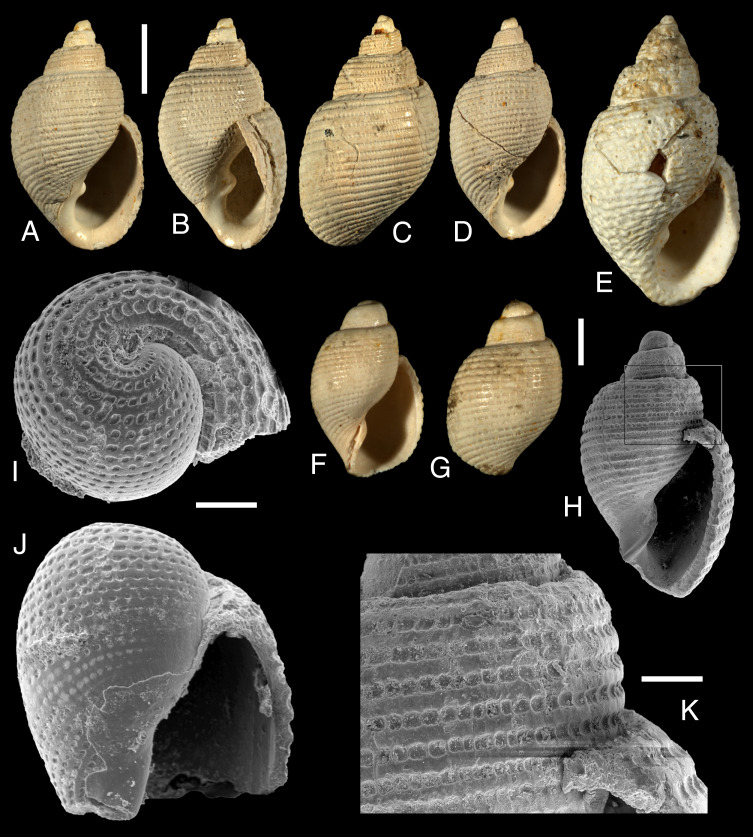
*Lanayrella vagabunda* (Mabille, 1885) from Monte Leon Formation (lower Miocene), Santa Cruz Province. (A–C) Three views of the shell (MACN-Pi 6938a). (D) View of the shell (MACN-Pi 6938b). (E) View of the shell (MACN-Pi 6938c). (F, G) Two views of a small specimen shell (MACN-Pi 6939a). (H) View of the shell at SEM (MACN-Pi 6938d). (I, J) Two views of the apical whorls at SEM (MACN-Pi 6938e). (K) Detail of the suture and sculpture at SEM (MACN-Pi 6939d). Scale bars: (A–E) two mm. (F–H) 500 µm. (I, J) 100 µm. (K) 200 µm.

Remarks: The type material of *L. ringei* was properly illustrated by Salvador & Cunha (2020). *Lanayrella ringei* is characterized by a shell sculptured with narrow spiral furrows and few growth lines (Fig. 1J), a diagnostic pattern that contrasts with all other species of *Lanayrella*, which possess wider spiral furrows and a greater number of growth lines (*e.g.*, Fig. 3K). Reports of Castellanos, Landoni & Dadon (1993) and Forcelli (2000) of *L. vagabunda* partially covered *L. ringei* because both species were considered as synonyms; however we are in agreement with Salvador & Cunha (2020) who demonstrated that they are distinct species. Forcelli & Narosky (2015) illustrated a specimen of *L. ringei* under the name *L. vagabunda*. The presence of lateral teeth of the radula is considered doubtful. Clearly delimited, relatively large, plate-like areas are visible on both sides of each rachidian tooth (Figs. 1K, 1L); however it is unclear whether these structures represent true lateral teeth or instead correspond to another layer that appears tooth-like due to folding over the rachidian during specimen fixation.

**Table:** 

*Lanayrella vagabunda* (Mabille, 1885)
Fig. 3

*Tornatella vagabunda* Mabille, 1885: 208; Rochebrune & Mabille, 1889: 12, pl. 6, figs 2a, b; Valdés & Héros, 1998: 698, fig 1C; Petit, 2010: 51

*Acteon vagabunda* Mabille & Rochebrune *sic*—Pilsbry, 1893: 164, pl. 18, figs 95, 96

*Actaeon vagabundus* (Mabille & Rochebrune)—Thiele, 1912: 265

*Acteon vagabunda* Mabille & Rochebrune, 1885 *sic*—Powell, 1960: 161

¿*Toledonia vagabunda* (Mabille, 1885)—Marcus, 1976: 27, fig24

*Acteon vagabundus* (Rochebrune & Mabille, 1885)—Castellanos, Landoni & Dadon, 1993: 8 (in part); Forcelli, 2000: 116 (in part)

*Acteon vagabundus* (Mabille & Rochebrune, 1885)—Linse, 1999: 403

*Lanayrella vagabunda* (Mabille, 1885)—Salvador & Cunha, 2020: 1011, fig 1

Type material: Syntype (MNHN-IM-2000-23191) from Cape Horn, Chile.

Examined material: Recent: Type material (photographs); Fossil: Cabeza de León (MACN-Pi 6938: 10 sh.); Monte Entrada (MACN-Pi 6939: 22 sh.).

Description: Shell (Figs. 3A–3H) small (up to 8.4 mm in length), thick, ovate to ovate-elongate, with 5½  whorls. Spire medium-sized about 25% of total length. Apical whorl (Figs. 3I, 3J) tilted, with slightly sunken nucleus; proto-teleoconch limit not visible. Teleoconch whorls convex, suture impressed to slightly channeled (Fig. 3K). Sculpture (Fig. 3K) consisting of wide, deep spiral furrows, regularly arranged over the entire shell surface: up to 11 on the apical whorl, five to seven on spire and 21 to 23 on the last whorl. Furrows are regularly interrupted by numerous, thin, slightly prosocline growth lines; intersections between furrows and lines produce a regular pitted pattern, more evident on early whorls (Figs. 3I, 3J). Aperture large, somewhat elongate; lip thin and sharp; columella strongly curved, abapically arched, with a single strong, fold; callus narrow.

Distribution: Recent: Cape Horn (type locality), Chile. Fossil: Cabeza de León and Monte Entrada localities, Santa Cruz province, Argentina. Samples come from Punta Entrada Member of Monte León Formation (lower Miocene).

Remarks: The type material of *L. vagabunda* was properly illustrated by Valdés & Héros (1998) and Salvador & Cunha (2020). Additional material reported here (Fig. 3) was collected from the Monte León Formation (lower Miocene) and represents the first fossil record of the genus. This material reveals variability in shell morphology, including elongated and more spired shells (cf. Figs. 3A–3C *vs.* 3D).

## Discussion

### *Lanayrella* diversity as an ancient endemic lineage of southern South America

The genus *Lanayrella* originally included *L. vagabunda* and *L. ringei*, which differ in their patterns of spiral sculpture: *L. vagabunda* has wider furrows, interrupted by more growth lines, whereas *L. ringei* has narrower furrows and fewer lines (cf. Figs. 3K *vs.* 1J). Knowledge of radular and anatomical characters, now available for *L. ringei* (Figs. 1D, 1K, 1L; 2), indicates that a third species of *Lanayrella* should be recognized based on the material studied by Marcus (1976) under the name *Toledonia bullata* (Gould, 1847). Accordingly, *Lanayrella bullata* n. comb. (Gould, 1847) is formally proposed here. Comparison of the three *Lanayrella* species shows overall similarity in shell morphology, whereas *L. ringei* and *L. bullata* n. comb. share several additional affinities in radular and anatomical characters. Shells of *L. bullata* n. comb. are very similar to those of *L. vagabunda,* both having wide spiral furrows interrupted by thin growth lines, although the former has fewer furrows (16 *vs.* 21 to 23 in the last whorl) with fewer lines (Gould, 1847; Gould, 1852–1856; Pilsbry, 1893; Marcus, 1976). The radulae of *L. bullata* n. comb and *L. ringei* are comparable, both showing a prominent, triangular rachidian tooth with few lateral cusps and slightly retracted central cusp. Marcus (1976) indicated the presence of relatively large, plate-like lateral teeth with several cusps in *L. bullata* n. comb., which seems consistent with the relatively large, plate-like areas observed in *L. ringei*, although in the latter no cusps are present (cf. Figs. 1K, 1L *vs.* Marcus (1976: left side of fig. 4)). It is not clear whether these lateral areas represent true lateral teeth or another layer reflecting the rachidian structure, possibly as a result of folding during specimen fixation. External anatomy further supports affinities between these two species. Both lack an operculum, the foot is notched anteriorly with several pedal glands opening into a ventral furrow; the cephalic shield is divided into two lobes; a pair of tentacular lobes is located just posterior to the cephalic shield; a large posterior lobe (= infrapallial lobe *sensu* Marcus, 1976) is present; the mantle bears an elongated kidney and gill positioned medially to posteriorly on the dorsal surface; and two repugnatory (?) glands are present, one anteriorly on the left side and the other posteriorly toward the right-dorsal side.

*Lanayrella* is here revealed as an ancient lineage of gastropods endemic to the Magellanic Region. The ocurrence of *L. vagabunda* in Miocene beds of the Monte León Formation (Fig. 3) represents the first fossil record of the genus, indicating that *Lanayrella* species have inhabited the Magellanic Region for at least ∼20 millon years.

The stratigraphic and geographic distribution of *Lanayrella* resembles patterns reported for other molluscan groups, particularly very small species (Griffin & Pastorino, 2012; Pastorino & Griffin, 2018; Pastorino & Griffin, 2019; Di Luca, Griffin & Pastorino, 2023). However, this pattern is not universal: most of the fauna from the Monte León Formation were adapted to warmer conditions and are absent from the cold environments currently prevailing in the Magellanic Region (del Río, 2004a; del Río, 2004b). Climatic changes in southern South America are associated with the final breakup of Gondwana, marked by the separation of Antarctica from South America, the opening of the Drake Passage, and the onset of the Antarctic Circumpolar Current, which began in the Eocene and established the cold climate characteristic of the Magellanic Region today (Violante et al., 2017).

The number of fossil and Recent specimens studied here, as well as those available from previous reports (Marcus, 1976; Castellanos, Landoni & Dadon, 1993; Forcelli, 2000; Forcelli & Narosky, 2015; Salvador & Cunha, 2020), suggest that *Lanayrella* species are rare. This rarity likely reflects naturally small population sizes rather than limitations in sampling. Understanding the ecological conditions in which *Lanayrella* species have lived and continue to live is essential; however, little is known about their diet, habitat, or reproduction. A direct developmental mode can be inferred from the relatively large, few-whorled protoconch with a large nucleus, indicating that juveniles hatch from the spawn without a free swimming larval stage. This reproductive strategy appears to be common among other gastropods of the Magellanic Region (*e.g.*, Di Luca, Penchaszadeh & Pastorino, 2020; Di Luca, Penchaszadeh & Pastorino, 2024; Di Luca & Pastorino, 2025).

### *Lanayrella* as a distinctive genus of cephalaspidean gastropods

The genus *Lanayrella* exhibits a unique combination of shell and anatomical characters that support its placement among cephalaspidean gastropods, in agreement with Marcus (1976). Nevertheless, Salvador & Cunha (2020) placed the genus within the Acteonidae. Among shell characters, only the protoconch morphology reliably distinguishes *Lanayrella* from *Acteon*. In *Lanayrella*, the transition between the protoconch and teleoconch is indistinct, and species are characterized by spiral furrows interrupted by fine growth lines which produce a regularly pitted pattern (Figs. 1G–1I; 3K). In contrast, *Acteon* species have a smooth protoconch which contrasts with a sculptured teleoconch (*e.g.*, Valdés, 2008). Thus, protoconch morphology results useful to recognize species of *Lanayrella* compared to those of *Acteon* and this is crucial in absence of soft parts, as occurs with the fossil material (*e.g.*, Fig. 3).

Soft anatomical and radular characters provide further evidence for affinities with cephalaspideans. As observed by Marcus (1976) and confirmed in the present study, *Lanayrella* lacks an operculum whereas it is present in Acteonidae (*e.g.*, Fretter & Graham, 1954; Mikkelsen, 1996; Mikkelsen, 2002; Burn & Thompson, 1998; Zelaya, Schejter & Ituarte, 2011). Radular morphology is highly variable within Acteonidae. The type of *Acteon*, *A. tornatilis* (Linné, 1758) and several other species bear rows of small, low, isolated denticles that are difficult to classify as rachidian, lateral, or marginal teeth (Fretter & Graham, 1954; Mikkelsen, 2002; Zelaya, Schejter & Ituarte, 2011). Other species can have elongated, cusped lateral/marginal teeth and typically lack a rachidian tooth (*e.g.*, Rudman, 1971; Marcus, 1974; Valdés, 2008). However, exceptions do occur such as the genus *Bathyacteon* Valdés, 2008, which has a relatively low and small rachidian tooth. In contrast, *Lanayrella* possesses a triangular, cusped rachidian that appears very prominent relative to its plate-like lateral areas (Fig. 1K, 1L), clearly differing from the radular morphologies reported for Acteonidae species.

### Affinities with *Toledonia* and *Bogasonia*

The systematic placement of *Lanayrella* within Cephalaspidea remains complex. Its spired, thick and wide shell, fully sculptured with spiral furrows, has no direct equivalent among other cephalaspidean taxa. Available evidence suggests that *Lanayrella* shares affinities with *Toledonia* and *Bogasonia,* both belonging to Toledoniinae Warén, 1989.

For example, *T. perplexa* Dall, 1902 and *T. globosa* Hedley, 1916 possess spired, broad shells with truncated columella, although the columellar fold is smaller than in *Lanayrella*. *Toledonia biplicata* (Strebel, 1908) has similarly strong columellar folds -up to three- whereas *Lanayrella* always bears a single one. Apical whorls are dextral and tilted in *Lanayrella*, *Toledonia* and *Bogasonia* all with a sunken nucleus and lacking a clear proto-teleoconch boundary (*e.g.*, Di Luca, Penchaszadeh & Pastorino, 2020; Di Luca & Pastorino, 2025), except in *T. warenella* Golding, 2010, where a faint mark is visible (Golding, 2010: fig. 1D). Spiral sculpture is strong throughout the shell in *Lanayrella*, whereas in *Toledonia* species it is often restricted to the initial whorls (*e.g.*, *T. biplicata*, *T. succinaeformis* Powell, 1955, *T. warenella*). The shell of *Lanayrella* is also distinctive in size, reaching more than 10 mm in height (Salvador & Cunha, 2020), whereas *Toledonia* and *Bogasonia* rarely exceed five mm (*e.g.*, *T. parelata* Dell, 1990, *B. gorjachevi* Chaban, 1998). In addition, the shells of the latter two genera are comparatively thinner, and their sculpture -when present- is mainly spiral, with poorly developed axial elements and mostly restricted to the apical whorls.

Radular and general morphological features of *Lanayrella* also resemble those of *Toledonia* and *Bogasonia*. The triangular, cusped rachidian is consistent in *Toledonia*, although the lateral plates are variable (Thiele, 1912; Odhner, 1914; Marcus, 1976; Linse, 2002; Valdés, 2008; Golding, 2010; Ohneiser & Malaquias, 2014). Some *Bogasonia* species display a similar rachidian with slightly retracted central cusp, but their lateral plates are smaller than in *Lanayrella* (Warén, 1989; Di Luca & Pastorino, 2025).

Anatomical features shared by *Lanayrella*, *Toledonia* and *Bogasonia* include a bilobed cephalic shield with large eyes at the base and a ventrally furrowed foot (Thiele, 1912; Chaban, 1998; Golding, 2010; Ohneiser & Malaquias, 2014; Di Luca & Pastorino, 2025). However, the cephalic shield lobes are smaller in *Toledonia* and *Bogasonia*. The posterior mantle lobe of *Lanayrella* (infrapallial lobe *sensu* Marcus, 1976) is considerably larger than in the other two genera (Golding, 2010; Di Luca & Pastorino, 2025). Other similarities include the presence of two large repugnatory (?) glands, an elongated kidney, and a posteriorly extended gill -features also observed in *T. limnaeaeformis* Smith, 1877 (Thiele, 1904, as *Odostomiopsis typica*). In *Bogasonia maradoniana* Di Luca & Pastorino, 2025 repugnatory (?) glands occur as small dark spots rather than the single large structure found in *Lanayrella*. Lateral tentacular lobes near the cephalic shield are present in *Lanayrella* and *T. warenella* (Golding, 2010) but absent in other *Toledonia* and *Bogasonia*. The hermaphroditic reproductive system of *Lanayrella*, with male gonopore near the right eye and female one on the right posterior side of the mantle, closely resembles those of *Toledonia* and *Bogasonia* (*e.g.*, Thiele, 1904; Chaban, 1998; Golding, 2010; Di Luca & Pastorino, 2025). A relatively simple, retractile penis occurs in the three genera (Chaban, 1998; Golding, 2010; Ohneiser & Malaquias, 2014; Di Luca & Pastorino, 2025), and at least one, prominent gland, possibly involved in capsules and nutritive liquid production, is present in many species (Chaban, 1998; Di Luca & Pastorino, 2025).

### Evolutionary and systematic implications

The affinities among *Lanayrella*, *Toledonia* and *Bogasonia* must be viewed in the context of cephalaspidean evolution. Their systematic positions remain controversial. Historically, *Toledonia*, *Bogasonia* and other genera (*e.g.*, *Newnesia* Smith, 1902) were placed in Diaphanidae (Marcus, 1976; Warén, 1989; Dell, 1990; Golding, 2010; Ohneiser & Malaquias, 2014) following Odhner’s (1914, 1926) classification. Toledoniinae Warén, 1989 was also proposed under Diaphanidae to include *Toledonia* and *Bogasonia*. However, later authors (*e.g.*, Jensen, 1996; Schiøtte, 1998; Golding, 2010; Ohneiser & Malaquias, 2014) questioned the monophyly of Diaphanidae, suggesting that many shared characters resulted from convergent evolution. Molecular phylogenies (Oskars, Bouchet & Malaquias, 2015; Oskars et al., 2019; Moles et al., 2017; Moles, Ávila & Malaquias, 2018) further revised this framework splitting the traditional Diaphanidae into several families (*e.g.*, Collinatydidiae Oskars, Bouchet & Malaquias, 2015, and Newnesiidae Moles et al., 2017). Unfortunately, these studies provide limited insight for *Lanayrella* because no species of the genus (and only one *Toledonia*) were included. Interestingly, *Toledonia* nested between two *Cylichna* Lovén, 1846 s.s. species, despite marked differences in shell and radular morphology as Oskars, Bouchet & Malaquias (2015) indicated. Following molecular evidence, *Toledonia* and the allied *Bogasonia* are now generally placed in Cylichnidae Adams & Adams, 1854 (*e.g.*, Bouchet et al., 2017; Valdés, 2019; Di Luca & Pastorino, 2025). *Lanayrella* shares with *Roxania* Leach, 1847 (Cylichnidae according to (Oskars, Bouchet & Malaquias, 2015) but later considered in Alacuppidae Oskars, Bouchet & Malaquias, 2015 by Oskars et al., 2019) a thick shell with strong spiral sculpture interrupted by fine growth lines. Similarities with Newnesiidae are also evident: the shell sculpture of spiral furrows interrupted by growth lines, a triangular rachidian, up to two plate-like laterals, and the presence of two repugnatory glands in a similar position (Moles et al., 2017; Moles, Ávila & Malaquias, 2018). However, Newnesiidae differs in several features as larger shell size (16.5 to 38 mm), thinner shells, partially exposed soft parts when they are retracted, mantle partially covering part of the shell in some species; triangular lateral plates, and a broader cephalic shield.

In this scenario, the family-level placement of *Lanayrella* is considered as uncertain. Affinities with *Toledonia*, as originally suggested by Marcus (1976) and corroborated here in addition with *Bogasonia*, support a position among Toledoniinae Warén, 1989 (also ranked as Toledoniidae by Kantor & Sysoev, 2006). In contrast, and according to recent molecular evidence (Oskars, Bouchet & Malaquias, 2015; Oskars et al., 2019; Moles et al., 2017; Moles, Ávila & Malaquias, 2018), *Toledonia* placed between two *Cylichna* s.s. species which implies that *Toledonia* (and any family placement derived from it) are part of the last genus even when several shell, radular and morphological differences are evident (Oskars, Bouchet & Malaquias, 2015). Also, Toledoniinae was listed among Cylichnidae by Bouchet et al. (2017). Additional molecular studies on *Toledonia*, *Bogasonia*, *Lanayrella* and other cephalaspidean gastropods are required to clarify the inner systematic of Cephalaspidea and thus the family placement of *Lanayrella*.

Finally the discovery of early Miocene specimens of *L. vagabunda* demonstrates the ancient origin of the genus. Many cephalaspidean gastropods are worldwide known from shells preserved in Miocene beds or even older (*e.g.*, Beu & Maxwell, 1990; Landau et al., 2013). However, very few of them seem to be comparable with *Lanayrella* by have highly distinctive shell characters to ensure a generic placement; furthermore this occurs in a scenario into which many of the traditional cephalaspidean taxa, as Cylichnidae and Diaphanidae, were split into many other families revealing that external similarities are due to convergent evolution (Oskars, Bouchet & Malaquias, 2015; Oskars et al., 2019). No *Toledonia* or *Bogasonia* species have been reported from the early Miocene; indeed, only one valid *Toledonia* species has been reported from Pleistocene deposits in Sweden (De Geer, 1910; Odhner, 1913; Marcus, 1976). *Toledonia flensburgensis* (Weinbrecht, 1994), described from the upper Miocene of Germany, only superficially resembles the genus because it shows a sharply delimited protoconch–teleoconch transition, where a smooth surface abruptly changes to a pitted teleoconch.

## Conclusions

*Lanayrella* species can be recognized by their *Acteon*-like shells, which lack a distinct boundary between protoconch and teleoconch.

A third *Lanayrella* species, previously reported by Marcus (1976) as *Toledonia*, is here formally proposed as *L. bullata* n. comb.

*Lanayrella* represents an ancient Magellanic lineage of highly distinctive gastropods dated from at least 20 millions years.

The combination of a shell without protoconch-teleoconch boundary, a foot without operculum, and a cephalic shield divided into two lobes indicates that *Lanayrella* belongs to the Cephalaspidea.

Shared teleoconch features between *Lanayrella* and members of the Acteonidae appear to have been acquired independently along the evolution of the groups.

*Lanayrella* seems to be related to *Toledonia* and *Bogasonia*.

Further studies using molecular characters are required to accurately determine the familial placement of *Lanayrella* species.

## Additional Information and Declarations

### Data Availability

The following information was supplied regarding data availability: The Muséum national d’Histoire naturelle (MNHN) data is available at GBIF: MNHN, Chagnoux S (2025). The molluscs collection (IM) of the Muséum national d’Histoire naturelle (MNHN - Paris). Version 70.420. MNHN - Museum national d’Histoire naturelle. Occurrence dataset https://doi.org/10.15468/xgoxap accessed *via* GBIF.org on 2026-03-17. https://www.gbif.org/occurrence/1019692022. Specimen ZMH 12833 is reposited at the Zologisches Institut und Zoologisches Museum der Universität (ZMH). Curator Bernard Häusdorf (b.hausdorf@leibniz-lib.de). Specimens MACN-In 44876 and MACN-in 44877 are reposited at Museo Argentino de Ciencias Naturales “Bernardino Rivadavia” (MACN). Curators Mariela Romanelli (biomariela60@hotmail.com, biomariela60@gmail.com) and Gabriela Liuzzi (gliuzzi@macn.gov.ar). Specimens MACN-Pi 6938 and MACN-Pi 6939 are reposited at Museo Argentino de Ciencias Naturales “Bernardino Rivadavia” (MACN). Curator Claudia del Río (claudiajdelrio@gmail.com).
